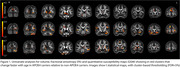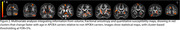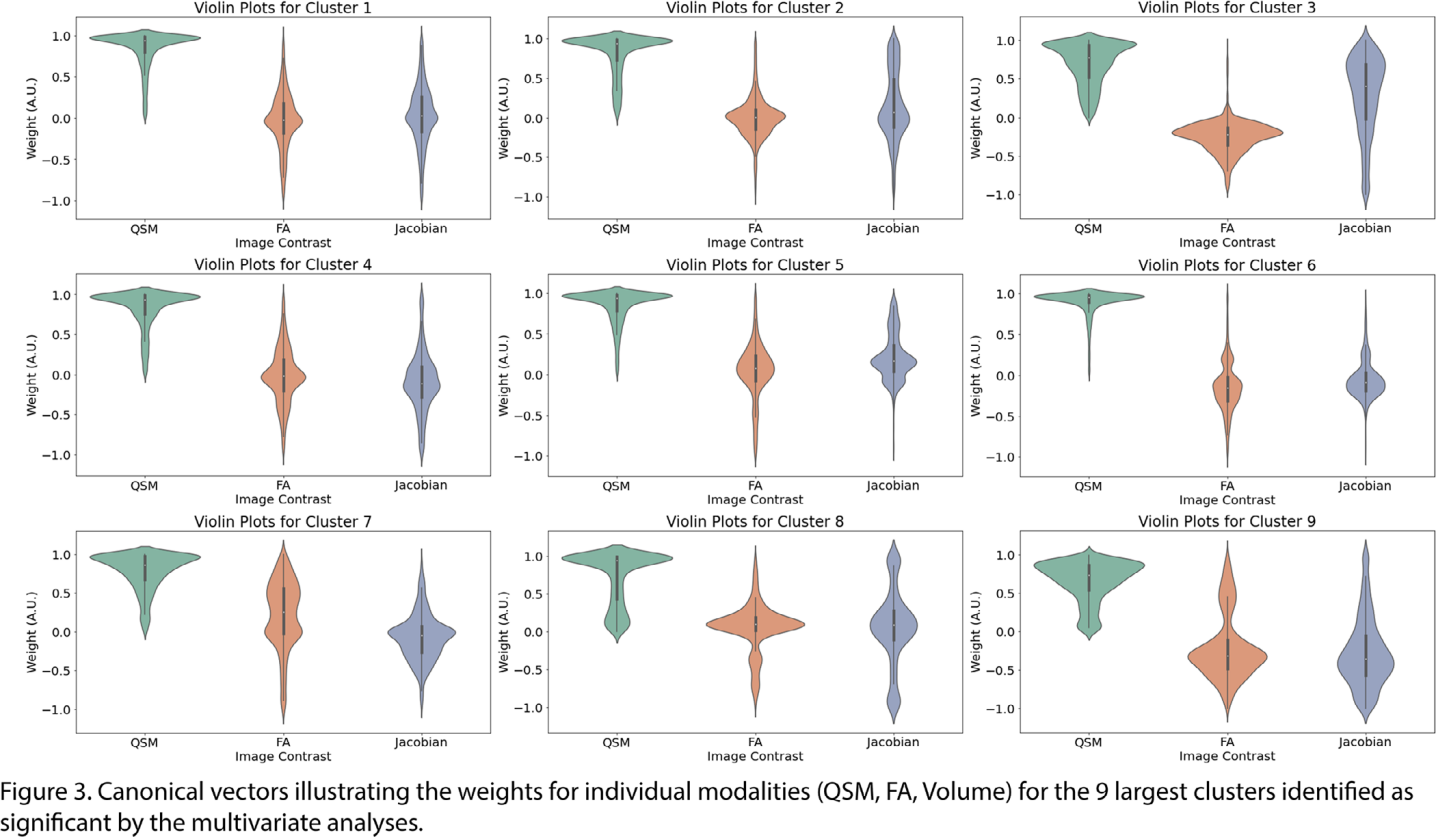# Multivariate Neuroimaging Analyses Reveal an Important Role for Iron Metabolism in Populations at Risk for Alzheimer’s Disease

**DOI:** 10.1002/alz.091934

**Published:** 2025-01-09

**Authors:** Alexandra Badea, Hae Sol Moon, Robert J Anderson, Ali Mahzarnia, Jacques Andrew Stout, Zay Yar Han, Kim G Johnson, Richard J O'Brien

**Affiliations:** ^1^ Duke University, Durham, NC USA

## Abstract

**Background:**

Alzheimer's disease (AD) is multifactorial, thus multivariate analyses help untangle its effects. We employed multiple contrast MRI to reveal age‐related brain changes in populations at risk for AD, due to APOE4 carriage. We assessed volume and microstructure changes using diffusion weighted imaging, and quantitative magnetic susceptibility maps (QSM) reflective primarily of cerebral iron metabolism.

**Method:**

Our study included 48 non APOE4 carriers, and 42 APOE4 carriers, with age ranging from 20.2 to 83; males and females. Imaging was done at 3T using diffusion weighted imaging (DWI) with TE 60.6 ms, TR=14725 ms, 21 diffusion directions (b=1000 s/mm2), reconstructed at 1 mm isotropic resolution using MUSE. A T1‐weighted FSPGR sequence was used for morphometry and co‐registration, TE=3.2 ms, TR=2263.7 ms, α=8°, prep time 900 ms, recovery time 700 ms, 1 mm isotropic resolution. QSM were calculated using STISuite based on fGRE images using TEeff=24.3 ms, TR 100 ms, α=15°, 8 echoes, reconstructed at 1x1x1.5 mm. Images were mapped into a common space before univariate and multivariate voxel‐based analyses (VBA).

**Result:**

Univariate VBA (Figure 1) showed accentuated age effects in APOE4 carriers in areas involved in visual information processing (pericalcarine and lingual cortex, precuneus); as well as in cognitive and motor control processes (superior frontal cortex). FA supported the findings in visual processing areas, and added regions in the left temporal lobe (entorhinal cortex, middle and inferior‐temporal cortices). FA also revealed a role for the anterior cingulate, insula, left cerebellum; pars triangularis and pars orbitalis, involved in language processing. QSM analyses supported the findings in visual, cingulate, and cerebellar cortices, and revealed a role for the hippocampus, amygdala, and basal ganglia (caudate, putamen, pallidum). Multivariate analyses (Figure 2) supported a role for the precuneus, cuneus, and lingual cortex, the inferior temporal and cingulate cortices, and the caudate. The canonical vectors (Figure 3) for the top significant clusters revealed an important role for QSM.

**Conclusion:**

Our results suggest accelerated changes in multiple domains besides memory in aging APOE4 carriers. Multivariate analyses supported an important role for age associated changes in cerebral iron metabolism, and oxidative stress, suggesting increased vulnerability to toxic insults.